# *Pyropia yezoensis* Protein Prevents Dexamethasone-Induced Myotube Atrophy in C2C12 Myotubes

**DOI:** 10.3390/md16120497

**Published:** 2018-12-08

**Authors:** Min-Kyeong Lee, Jeong-Wook Choi, Youn Hee Choi, Taek-Jeong Nam

**Affiliations:** 1 Institute of Fisheries Sciences, Pukyong National University, Busan 46041, Korea; 3633234@hanmail.net (M.-K.L.); wook8309@naver.com (J.-W.C.); unichoi@pknu.ac.kr (Y.H.C.); 2 Department of Marine Bio-Materials & Aquaculture, Pukyong National University, Busan 48513, Korea; 3 Department of Food Science and Nutrition, Pukyong National University, Busan 48513, Korea

**Keywords:** *Pyropia yezoensis*, protein, dexamethasone, muscle atrophy, forkhead box O, proteolytic system

## Abstract

Glucocorticoids (GCs), which are endocrine hormones released under stress conditions, can cause skeletal muscle atrophy. This study investigated whether *Pyropia yezoensis* crude protein (PYCP) inhibits synthetic GCs dexamethasone (DEX)-induced myotube atrophy associated with proteolytic systems. Mouse skeletal muscle C2C12 myotubes were treated with DEX in the presence or absence of PYCP. DEX exposure (100 μM) for 24 h significantly decreased myotube diameter and myogenin expression, which were all increased by treatment with 20 and 40 μg/mL PYCP. Additionally, PYCP significantly reduced the nuclear expression of the forkhead box transcription factors, FoxO1 and FoxO3a, and ubiquitin-proteasome pathway activation. Further mechanistic research revealed that PYCP inhibited the autophagy-lysosome pathway in DEX-induced C2C12 myotubes. These findings indicate that PYCP prevents DEX-induced myotube atrophy through the regulation of FoxO transcription factors, followed by the inhibition of the ubiquitin-proteasome and autophagy-lysosome pathways. Therefore, we suggest that inhibiting these two proteolytic processes with FoxO transcription factors is a promising strategy for preventing DEX-related myotube atrophy.

## 1. Introduction

Skeletal muscle accounts for more than 40% of the body and has important functions in metabolism, energy consumption, physical strength, and physical performance, and skeletal muscle mass is maintained by the relative balance of protein synthesis and degradation [1]. Skeletal muscle atrophy occurs when protein degradation exceeds protein synthesis as a result of long periods of rest, sedentary lifestyle, aging, starvation, and many pathological conditions (e.g., diabetes, cancer, HIV, sepsis, immune disorders, and kidney or heart failure) [2,3]. Among them, many pathological conditions are associated with increased circulating levels of glucocorticoids (GCs), which cause muscle atrophy. Recent studies have shown that sepsis-induced muscle atrophy is induced by a reduction in function of the cross-bridges between actin and myosin, which is at least partially regulated by GCs [4].

Although the mechanisms involved in GCs-induced skeletal muscle atrophy are not fully understood, some researchers have suggested that the inhibition of protein anabolism or stimulation of protein catabolism is responsible [5,6]. Previous studies have shown that GCs-induced skeletal muscle atrophy is mediated by the activation of major cellular proteolytic systems, such as the ubiquitin-proteasome system and the autophagy-lysosome system [7]. In the ubiquitin-proteasome system, ubiquitin causes protein degradation within the proteasome by binding to target proteins via three E1 (an ubiquitin-activating enzyme), E2 (an ubiquitin-conjugating enzyme), and E3 (an ubiquitin ligase) enzymes [8]. Two muscle-specific E3 ubiquitin ligases, atrogin-1/Muscle Atrophy F-Box (MAFbx) and muscle RING finger 1 (MuRF1), are reportedly involved in muscle protein degradation and are upregulated in muscle atrophy [9,10,11]. In previous studies, atrogin-1/MAFbx was reportedly increased 8–40-fold in muscle atrophy due to renal failure, cancer, and diabetes, and a 3-fold increase in muscle atrophy was induced by immobilization, denervation, and hindlimb suspension [9]. It has also been reported to be increased up to 10-fold in a cachectic or dexamethasone (DEX)-treated muscle atrophy model [10]. Furthermore, GCs increase the expression of the lysosomal protease cathepsin-L, leading to autophagy-lysosome-dependent muscle protein degradation [12,13]. Recent evidence indicates that the administration of DEX increases cathepsin-L and light chain 3 (LC3)-I/II expression in rat skeletal muscles [14,15]. These findings suggest that skeletal muscle atrophy induced by GCs exposure is mediated by the activation of the ubiquitin-proteasome and autophagy-lysosome systems.

Seaweeds are rich in minerals and various vitamins and are known to contain polysaccharides, proteins, lipids, and polyphenols [16,17]. They are an interesting potential source of food proteins, particularly owing to high protein levels and rich amino acid composition. The major seaweed species cultured in Korea are *Pyropia* spp., *Undaria* spp., and *Saccharina* spp. Among them, approximately 133 species of the marine red algae, *Pyropia* (Bangiales, Rhodophyta), have been reported from all over the world, including 28 species from Japan, 30 species from the North Atlantic coasts of Europe and America, and 27 from the Pacific coast of Canada [18]. *Pyropia* spp. include *P. tenera*, *P. yezoensis*, *P. suborbiculata*, and *P. dentate*. Among them, the species cultivated the most in Korea is *P. yezoensis* (Ueda) [19]. *P.yezoensis* is one of the most valuable seaweeds; it is widely cultivated in eastern Asian countries as well as Korea and has an annual global production of more than 1 million ton (fresh weight) and a market value of over US$ 1.5 billion per year [20]. In addition, *P. yezoensis* contains 25%–50% protein, 20%–40% polysaccharides, various vitamins, and essential minerals [21]. Moreover, it exerts various physiological effects, including anti-tumorigenic [22], anti-inflammatory [23], anti-photoaging [24], and anti-oxidant effects [25]. Previous studies reported that glycoproteins derived from *P. yezoensis* help reduce inflammatory stress induced by lipopolysaccharides in RAW264.7 cells and hepatotoxicity induced by ethanol in a rat model [23,26]. Moreover, *P. yezoensis* glycoproteins prevented cytotoxicity induced by D-galactosamine in Hepa 1c1c7 cells and acetaminophen-induced liver injury in a rat model [27,28]. These results imply that *P. yezoensis* is an effective natural substance for controlling various stress-related dysfunctions. 

Our previous study confirmed the protective effect of *P. yezoensis* peptides on dexamethasone (DEX)-induced myotube atrophy [29]. However, peptides are limited to oral ingestion due to chemical instability, such as degradation and aggregation of peptides by proteolytic enzymes present in the gastrointestinal tract. Therefore, in this study, we investigated the influence of *Pyropia yezoensis* crude protein (PYCP) on FoxO translocation and proteolytic systems in DEX-induced myotube atrophy.

## 2. Results

### 2.1. Effect of PYCP on Cell Viability

**Figure 1:**
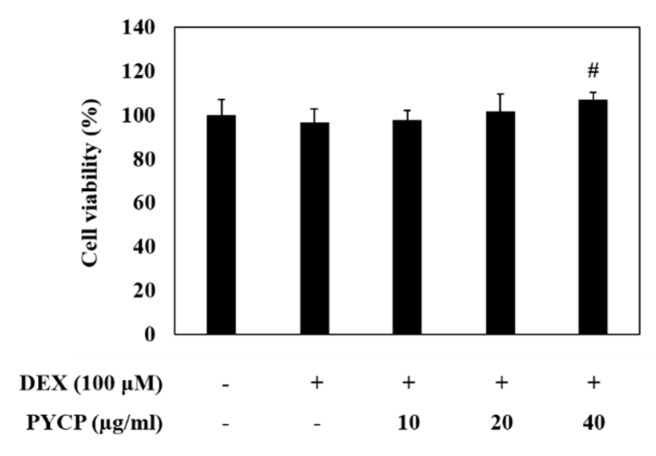
Effects of dexamethasone (DEX) and *Pyropia yezoensis* crude protein (PYCP) on the viability of mouse skeletal muscle C2C12 myotubes. Viability was determined using the 3-(4,5-dimethythiazol-2-yl)-5-(3-carboxymethoxyphenyl)-2-(4-sulfonyl)-2H-tetrazolium (MTS) assay, as described in the materials and methods section. The results are presented as the mean ± SD of three independent experiments. *^#^ p* < 0.05 vs. the corresponding DEX-only treatment group.

An MTS assay was performed to evaluate the cytotoxic effects of PYCP on C2C12 myotubes. C2C12 myotubes were incubated for 24 h with 100 μM DEX and PYCP at concentrations of 0 μg/mL to 40 μg/mL. The DEX (100 μM) dose was determined according to a previous study [29]. As shown in Figure 1, PYCP did not affect cell viability up to a concentration of 40 μg/mL. Thus, 100 μM DEX and 20 μg/mL or 40 μg/mL PYCP treatments were used to investigate the mechanism underlying the protective effects of PYCP against DEX-induced myotube atrophy in vitro.

### 2.2. PYCP Attenuates DEX-Induced Decreases in Myotube Diameter and Myogenin Expression in C2C12 Myotubes

**Figure 2:**
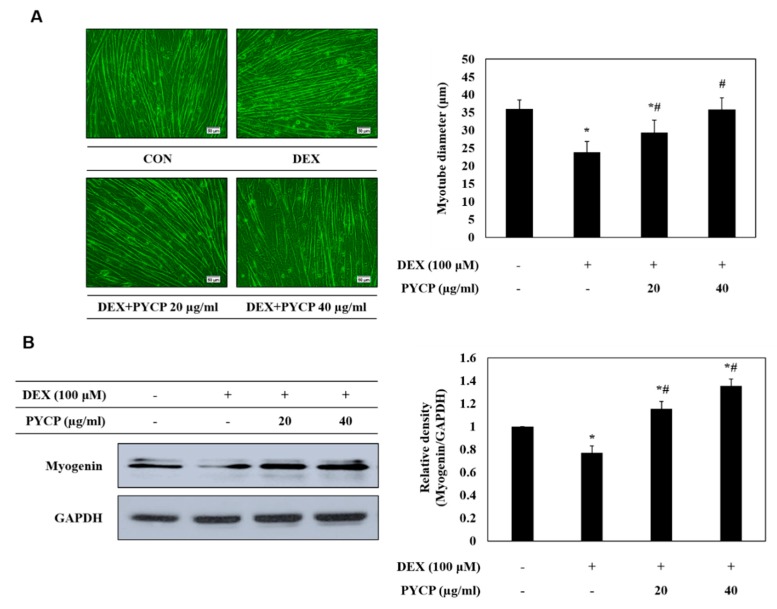
Effects of PYCP on myotube diameter and myogenin expression in DEX-stimulated C2C12 myotubes. (**A**) Representative images of C2C12 myotubes treated with 100 µM DEX and PYCP (20 μg/mL and 40 µg/mL). Comparison of myotube diameters among the four treatment groups. Images captured at ×20 magnification, scale bar represents 50 μm. (**B**) C2C12 myotubes were treated with 100 µM DEX and PYCP (20 μg/mL and 40 µg/mL) for 24 h. Myogenin protein levels were examined by Western blot analysis. Glyceraldehyde-3-phosphate dehydrogenase (GAPDH) was used as an internal standard. The results are presented as the mean ± SD of three independent experiments. * *p* < 0.05 vs. the corresponding control group; ^#^ *p* < 0.05 vs. the corresponding DEX-only treatment group.

To evaluate the effects of PYCP on myotube atrophy in C2C12 myotubes, cell diameter measurements were taken. C2C12 myotubes were allowed to differentiate for six days, followed by treatment with 100 μM DEX and 20 or 40 μg/mL PYCP for a further 24 h. As shown in Figure 2A, the DEX-treated group exhibited a 34% reduction in cell diameter compared with the control group, whereas the PYCP-treated group exhibited a dose-dependent increase in cell diameter. In addition, the expression levels of myogenin, a factor that regulates the terminal differentiation of muscle cells, were reduced in DEX-treated myotubes compared with controls. DEX-inhibited myogenin expression was markedly rescued by PYCP treatment at doses of 20 μg/mL and 40 μg/mL (Figure 2B).

### 2.3. PYCP Attenuates DEX-Induced Nuclear Translocation of FoxO1 and FoxO3a in C2C12 Myotubes

**Figure 3:**
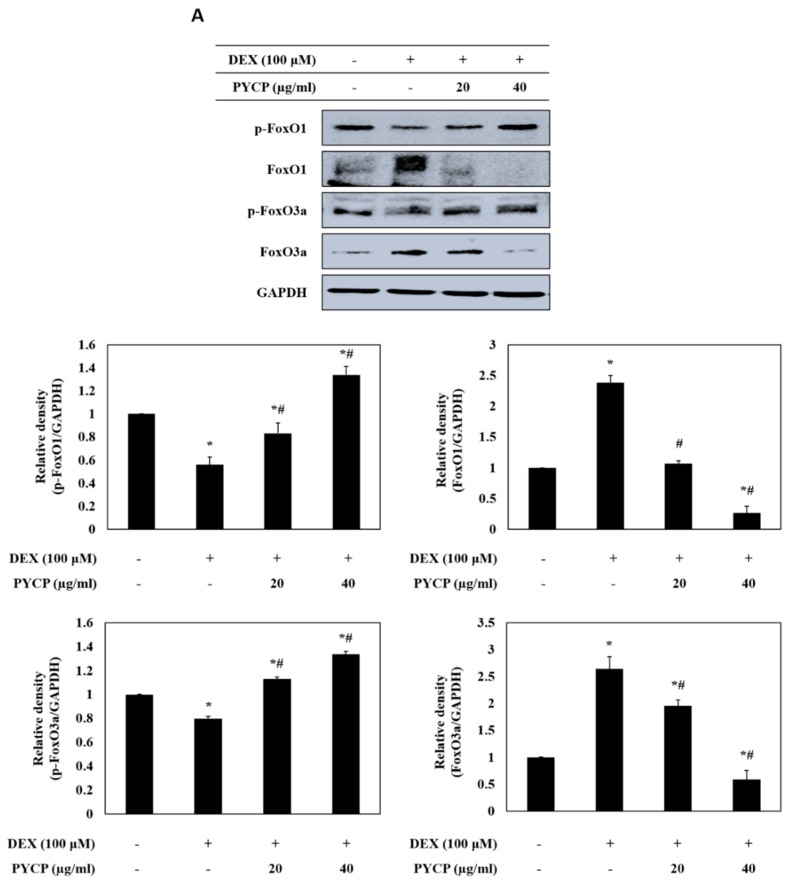

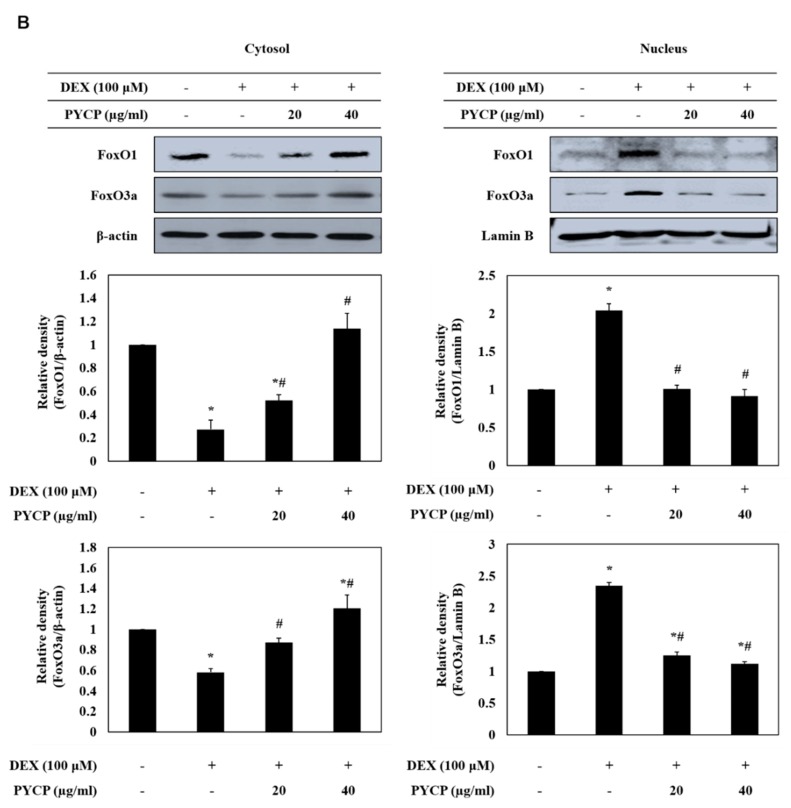
Effects of PYCP on the activation and translocation of FoxO1 and FoxO3a in DEX-stimulated C2C12 myotubes. C2C12 myotubes were treated with 100 µM DEX and PYCP (20 μg/mL and 40 µg/mL) for 24 h. (**A**) The protein levels of FoxO1 and FoxO3a were examined by Western blot analysis. (**B**) FoxO1 and FoxO3a levels were measured in cell cytosolic and nuclear fractions by Western blot analysis. GAPDH, β-actin, and lamin B were used as internal standards. The results are presented as the mean ± SD of three independent experiments. * *p* < 0.05 vs. the corresponding control group; ^#^ *p* < 0.05 vs. the corresponding DEX-only treatment group.

To determine whether PYCP inhibits the nuclear translocation of FoxO induced by DEX treatment, C2C12 myotubes were treated with DEX and PYCP for 24 h. As shown in Figure 3A, cells treated with DEX alone exhibited a marked increase in the protein levels of total FoxO1 and FoxO3a compared with cells in the untreated control group. However, the DEX-induced upregulation of FoxO1 and FoxO3a was attenuated by treatment with PYCP. In addition, the DEX-induced downregulation of p-FoxO1 and p-FoxO3a was attenuated by PYCP treatment. Inhibiting FoxO phosphorylation promotes the nuclear localization and transcriptional activity of FoxO [30]. The levels of nuclear FoxO1 and FoxO3a were significantly increased by DEX treatment, which was dose-dependently reduced by PYCP treatment (*p* < 0.05; Figure 3B). These results indicate that PYCP treatment effectively blocked the nuclear translocation and activation of FoxO by inhibiting DEX-induced FoxO dephosphorylation.

### 2.4. PYCP Attenuates DEX-Induced Increases in the Ubiquitin-Proteasome System in C2C12 Myotubes

**Figure 4:**
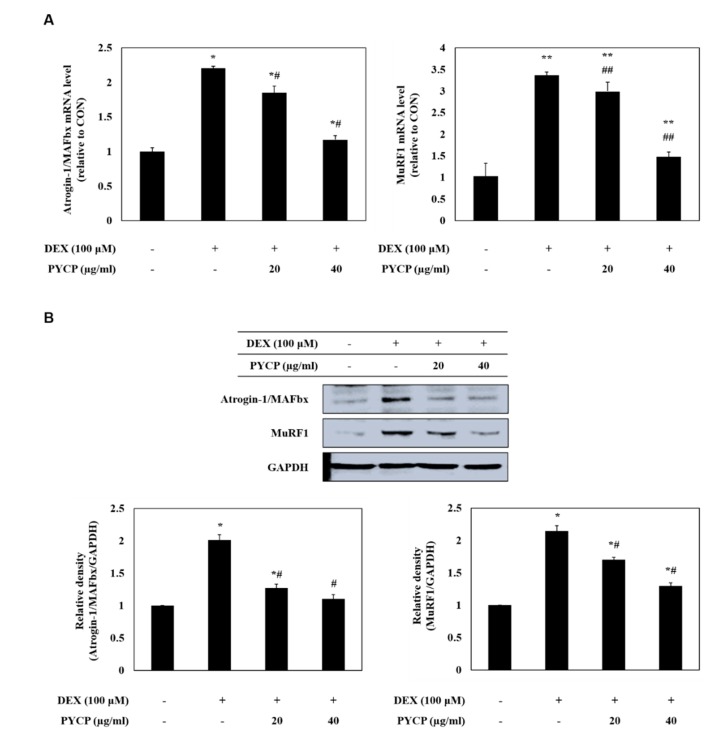
Effects of PYCP on mRNA and protein expression of E3 ubiquitin ligase in DEX-stimulated C2C12 myotubes. C2C12 myotubes were treated with 100 µM DEX and PYCP (20 μg/mL and 40 µg/mL) for 24 h. (**A**) The mRNA expression levels were quantified using real-time PCR. (**B**) The protein expression levels were measured using Western blot analysis. GAPDH was used as an internal standard. The results are presented as the mean ± SD of three independent experiments. * *p* < 0.05, ** *p* < 0.01 vs. the corresponding control group; ^#^ *p* < 0.05, ^##^ *p* < 0.01 vs. the corresponding DEX-only treatment group.

**Figure 5:**
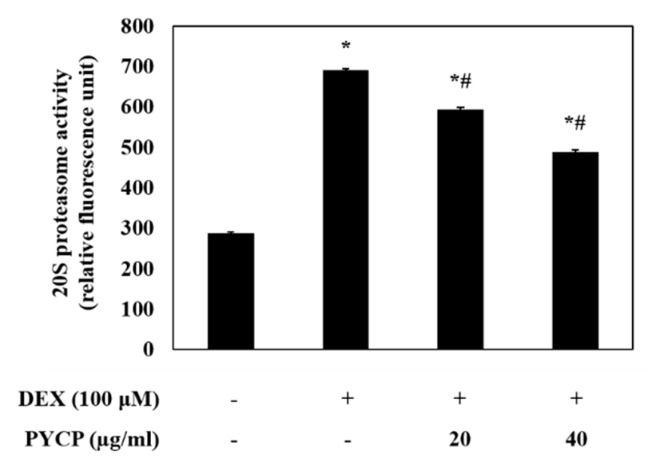
Effects of PYCP on 20S proteasome activity in DEX-stimulated C2C12 myotubes. 20S proteasomal activity was assessed by detecting 7-amino-4-methylcoumarin (AMC) in cell lysates after cleavage from the peptide LLVY-AMC. The results are presented as the mean ± SD of three independent experiments. * *p <* 0.05 vs. the corresponding control group; ^#^ *p <* 0.05 vs. the corresponding DEX-only treatment group.

To assess ubiquitin-proteasome system regulation by FoxO transcription factors, the mRNA and protein levels of atrogin-1/MAFbx and MuRF1 were measured in C2C12 myotubes. As shown in Figure 4, cells treated with DEX alone exhibited a marked increase in the mRNA and protein levels of atrogin-1/MAFbx and MuRF1 compared with cells in the untreated control group. However, the DEX-induced upregulation of atrogin-1/MAFbx and MuRF1 was attenuated by treatment with 20 μg/mL and 40 μg/mL PYCP. To explore the protective effects of PYCP on 20S proteasome activity further, we conducted 20S proteasome activity assays. As shown in Figure 5, 20S proteasome activity was distinctly enhanced by DEX treatment, whereas it was attenuated by PYCP treatment in a dose-dependent manner.

### 2.5. PYCP Attenuates DEX-Induced Increased Autophagy-Lysosome System Activity in C2C12 Myotubes

**Figure 6:**
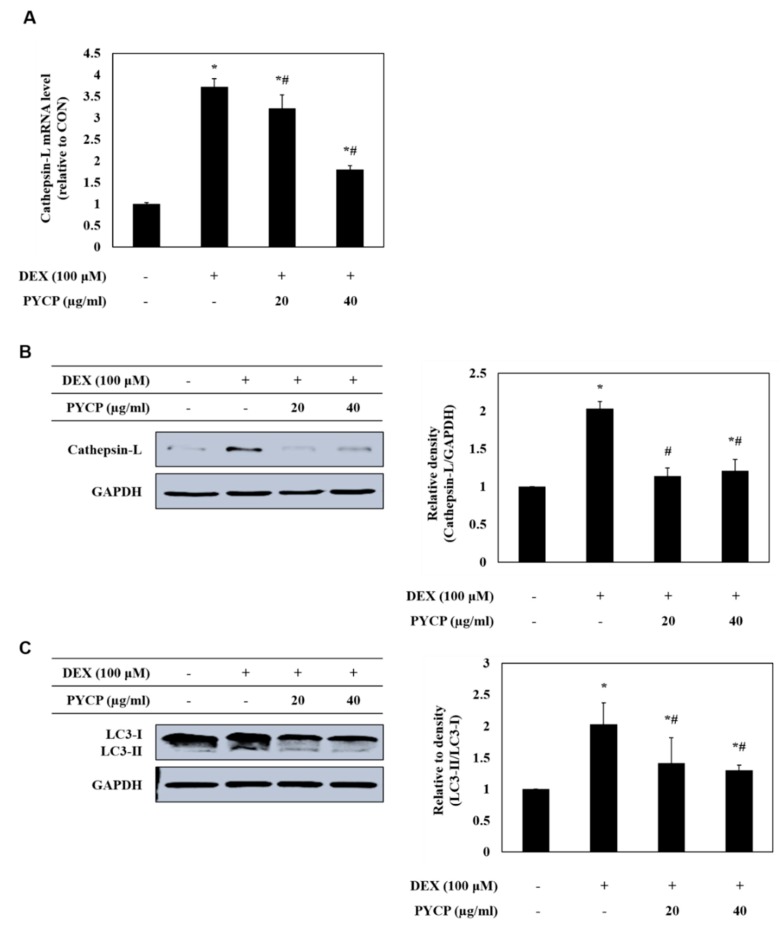
Effects of PYCP on mRNA and protein expression of cathepsin-L and LC3-I/II in DEX-stimulated C2C12 myotubes. C2C12 myotubes were treated with 100 µM DEX and PYCP (20 μg/mL and 40 µg/mL) for 24 h. (**A**) The mRNA level of cathepsin-L was quantified by real-time PCR. (**B**) The protein level of cathepsin-L was measured using Western blot analysis. (**C**) The protein level of LC3-I/II was measured using Western blot analysis. GAPDH was used as an internal standard. The results are presented as the mean ± SD of three independent experiments. * *p* < 0.05 vs. the corresponding control group; ^#^ *p* < 0.05 vs. the corresponding DEX-only treatment group.

To assess autophagy-lysosome system regulation by the FoxO transcription factors further, the mRNA and protein levels of cathepsin-L and LC3-I/II were measured in C2C12 myotubes. As shown in Figure 6A,B, DEX treatment significantly increased the mRNA and protein levels of cathepsin-L in C2C12 myotubes (*p* < 0.05). However, treatment with PYCP downregulated the DEX-induced increase in cathepsin-L. In addition, the DEX-induced conversion of LC3-I to LC3-II was downregulated by PYCP treatment in a dose-dependent manner (Figure 6C).

## 3. Discussion

GCs have been used to treat various chronic inflammatory diseases, such as rheumatoid arthritis [31], sarcoidosis [32], and bronchial asthma [33]. Many previous studies have shown that administering high doses of GCs results in skeletal muscle atrophy [34,35]. We investigated the effects of PYCP on skeletal muscle atrophy induced by DEX in an in vitro model.

Myogenin plays a dual role as a regulator of muscle development and as an inducer of neurogenic atrophy, such as denervation [36,37,38]. Recent studies have demonstrated that DEX reduces myotube diameter [39] and myogenin expression [36], thus leading to muscle atrophy. In particular, DEX reportedly induces the expression of glucocorticoid-induced leucine zipper (GILZ), thereby reducing the expression of myogenin [37]. Conversely, myogenin acts as an upstream inducer of E3 ubiquitin ligases in the denervation atrophy model [38]. Thus, myogenin can exert opposing effects, such as promoting differentiation or degradation, depending on developmental or pathological conditions in skeletal muscle. In the present study, the diameter of C2C12 myotubes decreased following DEX exposure, but was rescued by PYCP treatment. Similarly, the expression of myogenin, which was inhibited by DEX exposure, was upregulated by PYCP treatment. GCs can induce muscle atrophy by inhibiting muscle development through downregulation of myogenin expression, which is essential for satellite cell differentiation into muscle fibers [40]. Therefore, these results indicate that PYCP prevents muscle atrophy by promoting muscle development through the activation of myogenin expression suppressed by DEX exposure.

FoxO transcription factors regulate muscle atrophy and play an essential role in regulating the expression of atrogenes, such as atrogin-1/MAFbx, MuRF1, and cathepsin-L [41]. The FoxO family of transcription factor in skeletal muscles comprises three isoforms, FoxO1, FoxO3, and FoxO4, and the translocation of FoxO is regulated by Akt [42,43]. Previous studies have consistently reported that Akt blocks the upregulation of atrogin-1/MAFbx and MuRF1 by phosphorylating FoxO and inhibiting its translocation to the nucleus during myotube atrophy [36,44]. DEX-induced myotube atrophy results in reduced phosphorylation of Akt, FoxO1, and FoxO3a, and increased expression of atrogin-1/MAFbx and MuRF1 [44]. In addition, the overexpression of FoxO3 in L6 myotubes strongly activates expression of atrogin-1/MAFbx [45]. Therefore, we assessed the protein expression of two FoxO family members, FoxO1 and FoxO3a, following treatment with DEX and PYCP in C2C12 myotubes. Our results show that the expression of FoxO1 and Foxo3a was inhibited by PYCP treatment, resulting in decreased nuclear translocation. Several previous studies have shown that the DEX-induced increased expression of FoxO1 and FoxO3a leads to the activation of a gene transcription program that results in muscle atrophy [46]. Although the underlying mechanism is unclear, the ubiquitin-proteasome system is the proteolytic system currently considered the most important mechanism of muscle atrophy [7]. The protein targeted for degradation by the ubiquitin-proteasome system should be labeled with the covalent attachment of the ubiquitin molecular chain comprising 76 amino acids [47]. Ubiquitin is activated by three consecutive enzymes before binding the target protein and then induces the degradation of the target protein by the proteasome (20S or 26S) [48]. E3 ubiquitin ligase plays a crucial role in identifying and targeting proteins for proteasomal degradation [49,50]. Previous studies characterized two muscle-specific E3 ubiquitin ligases, atrogin-1/MAFbx and MuRF1, as markers of skeletal muscle atrophy [11]. Therefore, we determined the mRNA and protein expression of atrogin-1/MAFbx and MuRF1 in C2C12 myotubes after DEX and PYCP treatment. This data revealed that the DEX-stimulated expression of atrogin-1/MAFbx and MuRF1 was downregulated by PYCP treatment. Similarly, 20S proteasomal activity was enhanced by DEX treatment and downregulated by PYCP treatment. In addition, previous studies have confirmed that the autophagy-lysosome system is activated in muscle cells under catabolic conditions [14,51,52]. In skeletal muscles, DEX induces muscle atrophy by increasing the expression of cathepsin-L and promoting the conversion of LC3-I and LC3-II [53]. In this study, the DEX-induced expression of cathepsin-L and conversion of LC3-I to LC3-II was downregulated by PYCP treatment. These results reveal that PYCP prevented the DEX-induced myotube atrophy by downregulating the nuclear translocation of FoxO transcription factors and downregulating the ubiquitin-proteasome and autophagy-lysosome systems.

In conclusion, this study revealed that DEX plays a role in regulating FoxO transcription factors and activating the muscle atrophy-related E3 ubiquitin ligase, thus supporting the hypothesis that DEX exerts its pro-catabolic action via this pathway. In addition, the results provide molecular evidence that the anti-muscle atrophy effects of PYCP are at least partially regulated by FoxO transcription factors and reflect inhibited upregulation of the ubiquitin-proteasome and autophagy-lysosome systems.

## 4. Materials and Methods 

### 4.1. Preparation of PYCP

*P. yezoensis* was purchased in 2017 (Suhyup, Busan, Korea), washed several times with tap water to remove salt and visible epiphytes, and stored at −20 °C until use. *P. yezoensis* powder (40 g) was diluted with 1 L distilled water and stirred for 4 h at room temperature. The solution was centrifuged at 3000× *g* at 4 °C for 10 min, and vacuum filtered through a crucible. A 3 × volume of ethanol was added to the solution (total quantity of filtrate × 3). After 24 h, the solution was filtered and concentrated using rotary evaporation at 40 °C. The supernatant was added to 80% ammonium sulfate and stirred for 24 h at 4 °C. Salts were then removed through a 3500-Da MW Spectra/Pormembrane (Spectrum Labs, Rancho Dominguez, CA, USA) for 48 h at 4 °C. The resulting solution was dialyzed against distilled water and then concentrated. The concentrated solution was distributed into 1.5 mL tubes and freeze-dried to produce a powder. The powder was stored at −70 °C until use, and named PYCP. The PYCP extracts were solubilized with ddH_2_O for use in the assays.

### 4.2. Cell culture and Differentiation

C2C12 mouse skeletal muscle cells (ATCC CRL-1772; American Type Culture Collection, Manassas, VA, USA) were cultured in Dulbecco’s modified Eagle’s medium (DMEM; Gibco; Thermo Fisher Scientific, Waltham, MA, USA) supplemented with 10% fetal bovine serum (FBS; Gibco; Thermo Fisher Scientific), 100 U/mL penicillin, and 100 µg/mL streptomycin (Gibco; Thermo Fisher Scientific) at a temperature of 37 °C in a humidified atmosphere of 5% CO_2_. C2C12 myoblasts were grown to 70%–80% confluence in culture dishes (10 mm) at 37 °C, then trypsinized and seeded (4 × 10^4^ cells/well) into six-well culture plates for experiments. Cells were grown to 70%–80% confluence in DMEM supplemented with 10% FBS at 37 °C for 24 h, at which time the medium was replaced with DMEM containing 2% FBS to induce differentiation into myotubes, and the medium was replaced every 2 days. Cells were allowed to differentiate for 6 days, at which point 90% of the cells had fused into myotubes.

### 4.3. Treatment with DEX and PYCP

Following 6 days of differentiation, C2C12 myotubes were subdivided into four groups: The control group, in which cells were incubated in serum-free medium (SFM; DMEM containing 100 U/mL penicillin and 100 µg/mL streptomycin); the DEX group, in which cells were treated with 100 μM DEX; the DEX + PYCP group, in which cells were treated with 100 μM DEX and 20 μg/mL PYCP; and the DEX + PYCP group, in which cells were treated with 100 μM DEX and 40 μg/mL PYCP. All groups were incubated in serum-free medium at 37 °C for 24 h prior to harvesting cells for experiments.

### 4.4. MTS Assay

Cell viability was measured using the CellTiter 96 Aqueous Non-Radioactive Cell Proliferation Assay (Promega Corporation, Madison, WI, USA), which is based on the formation of a formazan product from tetrazolium compound MTS (3-(4,5-dimethylthiazol-2-yl)-5-(3-carboxymethoxyphenyl)-2-(4-sulfonyl)-2*H*-tetrazolium). Briefly, cells (1.5 × 10^4^ cells/well) were seeded in 96-well plates in 100 μL DMEM supplemented with 10% FBS and were allowed to attach at 37 °C for 24 h. After differentiation, the cells were incubated with 100 μM DEX and PYCP (10, 20, and 40 μg/mL) for 24 h at 37 °C. MTS solution (10 μL) was added and the cells were incubated at 37 °C for 30 min. The absorbance at 490 nm was measured using a Gen5 ELISA (Bio-Tex, Houston, TX, USA). Experiments were performed in triplicate.

### 4.5. Measurement of Myotube Diameters

Myotube cultures were photographed under a phase contrast microscope at 200 magnification after treatment with 100 μM DEX and 20 μg/mL and 40 μg/mL PYCP for 24 h. Myotube diameters were measured using a method of Hasselgren et al. [6,54,55]. The diameters were measured in a total of 50 myotubes from at least 10 random fields using Image J software (version 4.16; National Institutes of Health, Bethesda, MD, USA). The measurements were conducted in a “blinded” fashion with the researcher being unaware from which experimental group the cultures originated.

### 4.6. Real-Time Polymerase Chain Reaction

The mRNA expression levels of specific genes were evaluated using real-time PCR. Total RNA was isolated from C2C12 myotubes using TRIzol reagent (Invitrogen Life Technologies, Carlsbad, CA, USA). RNA concentration and purity were evaluated by determining the ratio of absorbance readings at 260 nm and 280 nm (A260/A280). A RevoScript Reverse Transcriptase PreMix Kit (Intron Biotechnology Co., Ltd., Seongnam, Korea) was used to prepare cDNA according to the manufacturer’s instructions, and the samples were stored at −50 °C. Real-time PCR was conducted in 20 μL reactions using a QuantiMix SYBR kit (PhilKorea Technology, Inc., Daejeon, Korea) and an Illumina Eco Real-Time PCR system (Illumina, Inc., Hayward, CA, USA). All mRNA levels were normalized using glyceraldehyde-3-phosphate dehydrogenase (GAPDH) as an internal control. The primers used for amplification are shown in Table 1.

### 4.7. Preparation of Total Cell Lysates

Cells were allowed to differentiate for 6 days at 37 °C, followed by incubation at 37 °C for 24 h in either SFM (control group) or SFM containing 100 μM DEX (DEX group), 100 μM DEX + 20 μg/mL PYCP (DEX + PYCP group), and 100 μM DEX + 40 μg/mL PYCP (DEX+PYCP group). Cells were washed with cold PBS and lysed with extraction buffer [1% NP-40, 0.25% sodium deoxycholate, 1 mM ethylene glycol-bis (β-aminoethyl ether)-*N*,*N*,*N*′,*N*′-tetraacetic acid, 150 mM NaCl, and 50 mM Tris-HCl, pH 7.5] containing protease inhibitors (1 mg/mL aprotinin, 1 mg/mL leupeptin, 1 mg/mL pepstatin A, 200 mM Na_3_VO_4_, 500 mM NaF, and 100 mM PMSF) on ice. Extracts were centrifuged at 16,000× *g* for 10 min at 4 °C, and protein levels were quantified using a bicinchoninic acid (BCA) protein assay kit (Pierce; Thermo Fisher Scientific) according to the manufacturer’s instructions. The supernatant was then used in Western blot analysis.

### 4.8. Preparation of Cytosolic and Nuclear Extracts

Cells were treated and harvested as described above, lysed with hypotonic lysis buffer [25 mM 4-(2-hydroxyethyl)-1-piperazineethanesulphonic acid (HEPES; pH 7.5), 5 mM EDTA, 5 mM MgCl_2_, and 5 mM dithiothreitol (DTT)], and incubated for 15 min on ice. NP-40 (2.5%) was added and the cells were lysed for an additional 10 min. Nuclei were collected by centrifugation at 7500× *g* for 15 min at 4 °C. The supernatant was collected as the cytosolic fraction. Nuclear proteins were resuspended in extraction buffer (10 mM HEPES, pH 7.9, 100 mM NaCl, 1.5 mM MgCl_2_, 0.1 mM EDTA, and 0.2 mM DTT) and incubated for 20 min at 4 °C. Extracts were centrifuged at 16,000× *g* for 10 min, and protein levels were determined using a BCA protein assay kit (Pierce; Thermo Fisher Scientific, Inc.) according to the manufacturer’s instructions. Both fractions were then used for Western blot analysis.

### 4.9. Western Blot Analysis

Equal amounts of proteins (30 μg) were separated by 6%–12.5% sodium dodecyl sulfate-polyacrylamide gel electrophoresis and transferred to a polyvinylidene fluoride membrane (Millipore, Bedford, MA, USA). The membrane was blocked at room temperature with 1% bovine serum albumin (BSA) in TBS-T (10 mM Tris-HCl, 150 mM NaCl, and 0.1% Tween-20) and then incubated with primary antibodies (Table 2). The secondary antibodies (diluted 1:10,000–1: 20,000) were horseradish peroxidase-conjugated anti-rabbit IgG (7074S; Cell Signaling Technology, Inc., Beverly, MA, USA), donkey anti-goat IgG (A50-101P; Bethyl Laboratories, Inc., Montgomery, TX, USA), or goat anti-mouse IgG (sc-2031; Santa Cruz Biotechnology, Inc., Santa Cruz, CA, USA). Signals were detected using an enhanced chemiluminescence Western blot analysis kit (Thermo Fisher Scientific, Rockford, IL, USA). Experiments were performed in triplicate and densitometry analysis was performed using Multi-Gauge software version 3.0 (Fujifilm Life Science, Tokyo, Japan).

### 4.10. 20S Proteasome Activity Assay

The chymotrypsin-like activity of the 20S proteasome was measured by changes in the fluorescence of 7-amino-4-methylcoumarin (AMC) conjugated to the chymotrypsin peptide substrate LLVY, using a 20S proteasome activity assay kit (Chemicon, Temecula, CA, USA). In brief, cells were suspended in RIPA lysis buffer (50 mM Tris-HCl, pH 7.5, 150 mM sodium chloride, 0.5% sodium deoxycholate, 1% Triton X-100, 0.1% SDS, and 2 mM EDTA) containing protease inhibitors (1 mg/mL aprotinin, 1 mg/mL leupeptin, 1 mg/mL pepstatin A, 200 mM Na_3_VO_4_, 500 mM NaF, and 100 mM PMSF) and centrifuged at 16,000× *g* for 10 min at 4 °C. The protein concentration of supernatants was determined with a BCA protein assay (Pierce, Rockford, IL, USA). The cell lysates were incubated for 90 min at 37 °C with a labeled substrate, LLVY-AMC, and the cleavage activity was monitored by detecting the free fluorophore AMC using a fluorescence plate reader (Gen5 ELISA; Bio-Tek).

### 4.11. Statistical Analysis

Mean values were assessed by analysis of variance using SPSS version 10.0 (SPSS Inc., Chicago, IL, USA). Values are presented as means ± standard deviation. Different letters indicate significant differences between groups, according to Duncan’s multiple-range test.

## Figures and Tables

**Table 1:** Oligonucleotide primer sequences used in real-time PCR.

Gene	Accession No.	Sequence (5′–3′)	Amplicon Size (bp)
Atrogin-1/MAFbx	NM_026346.3	F: ATGCACACTGGTGCAGAGAG R: TGTAAGCACACAGGCAGGTC	168
Cathepsin-L	M20495.1	F: GACCGGGACAACCACTGTG R: CCCATCAATTCACGACAGGAT	61
MuRF1	DQ229108.1	F: TGTCTGGAGGTCGTTTCCG R: GTGCCGGTCCATGATCACTT	59
GAPDH	NM_008084.3	F: ACTCCACTCACGGCAAATTCA R: CGCTCCTGGAAGATGGTGAT	91

**Table 2:** Primary antibodies used in Western blot analysis.

Name of Antibody	Manufacturer and Catalog No.	Species Raised in Monoclonal or Polyclonal	Dilution Rate
Atrogin-1/MAFbx	Santa Cruz Biotechnology: sc-27645	Rabbit	1: 2000
β-actin	Santa Cruz Biotechnology: sc-47778	Mouse	1: 1000
Cathepsin-L	Santa Cruz Biotechnology: sc-6498	Rabbit	1: 1000
FoxO1	Santa Cruz Biotechnology: sc-374427	Mouse	1: 500
FoxO3a	Santa Cruz Biotechnology: sc-9813	Rabbit	1: 1000
GAPDH	Santa Cruz Biotechnology: sc-25778	Rabbit	1: 1000
Lamin B	Santa Cruz Biotechnology: sc-377000	Rabbit	1: 1000
LC3-I/II	Cell Signaling: #4108S	Rabbit	1: 1000
MuRF1	Santa Cruz Biotechnology: sc-27642	Goat	1: 2000
Myogenin	Santa Cruz Biotechnology: sc-12732	Rabbit	1: 1000
p-FoxO1	Cell Signaling: #9461S	Rabbit	1: 500
p-FoxO3a	Cell Signaling: #9466S	Rabbit	1: 1000

## References

[1] GGFry C.S., Drummond M.J., Glynn E.L., Dickinson J.M., Gundermann D.M., Timmerman K.L., Walker D.K., Dhanani S., Volpi E., Rasmussen B.B. (2011). Aging impairs contraction-induced human skeletal muscle mTORC1 signaling and protein synthesis. Skelet. Muscle.

[2] Cruz-Jentoft A.J., Baeyens J.P., Bauer J.M., Boirie Y., Cederholm T., Landi F., Martin F.C., Michel J.P., Rolland Y., Vandewoude M. (2010). Sarcopenia: European consensus on definition and diagnosis: Report of the European working group on sarcopenia in older people. Age Ageing.

[3] Wang Y., Pessin J.E. (2013). Mechanisms of fiber-type specificity of skeletal muscle atrophy. Curr. Opin. Clin. Nutr. Metab. Care.

[4] Alamdari N., Toraldo G., Aversa Z., Smith I., Castillero E., Renaud G., Qaisar R., Hasselgren P.O. (2012). Loss of muscle strength during sepsis is in part regulated by glucocorticoids and is associated with reduced muscle fiber stiffness. Am. J. Physiol. Regul. Integr. Comp. Physiol..

[5] Baehr L.M., Furlow J.D., Bodine S.C. (2011). Muscle sparing in muscle RING finger 1 null mice: Response to synthetic glucocorticoids. J. Physiol..

[6] Castillero E., Alamdari N., Lecker S.H., Hasselgren P.O. (2013). Suppression of atrogin-1 and MuRF1 prevents dexamethasone-induced atrophy of cultured myotubes. Metabolism.

[7] Hasselgren P.O. (1999). Glucocorticoids and muscle catabolism. Curr. Opin. Clin. Nutr. Metab. Care.

[8] Lecker S.H., Goldberg A.L., Mitch W.E. (2006). Protein degradation by the ubiquitin-proteasome pathway in normal and disease states. J. Am. Soc. Nephrol..

[9] Bodine S.C., Latres E., Baumhueter S., Lai V.K., Nunez L., Clarke B.A., Poueymirou W.T., Panato F.J., Na E., Dharmarahan K. (2001). Identification of ubiquitin ligases required for skeletal muscle atrophy. Science.

[10] Gomes M.D., Lecker S.H., Jagoe R.T., Navon A., Goldberg A.L. (2001). Atrogin-1, a muscle-specific F-box protein highly expressed during muscle atrophy. Proc. Natl. Acad. Sci. USA.

[11] Lecker S.H., Jagoe R.T., Gilbert A., Gomes M., Baracos V., Bailey J., Price S.R., Mitch W.E., Goldberg A.L. (2004). Multiple types of skeletal muscle atrophy involve a common program of changes in gene expression. FASEB J..

[12] Gilson H., Schakman O., Combaret L., Lause P., Grobet L., Attaix D., Ketelslegers J.M., Thissen J.P. (2007). Myostatin gene deletion prevents glucocorticoid-induced muscle atrophy. Endocrinology.

[13] Schakman O., Kalista S., Barbe C., Loumaye A., Thissen J.P. (2013). Glucocorticoid-induced skeletal muscle atrophy. Int. J. Biochem. Cell Biol..

[14] Deval C., Mordier S., Obled C., Bechet D., Combaret L., Attaix D., Ferrara M. (2001). Identification of cathepsin L as a differentially expressed message associated with skeletal muscle wasting. Biochem. J..

[15] Yamamoto D., Maki T., Herningtyas E.H., Ikeshita N., Shibahara H., Sugiyama Y., Nakanishi S., Iida K., Iguchi G., Takahashi Y. (2010). Branched-chain amino acids protect against dexamethasone-induced soleus muscle atrophy in rats. Muscle Nerve.

[16] Arasaki S., Arasaki T. (1983). Low Calorie, High Nutrition: Vegetables from the Sea. To Help You Look and Feel Better.

[17] Kumar C.S., Ganesan P., Suresh P.V., Bhaskar N. (2008). Seaweeds as a source of nutritionally beneficial compounds-a review. J. Food Sci. Technol..

[18] Yoshida T., Notoya M., Kikuchi N., Miyata M. (1997). Catalogue of species of *Porphyra* in the world, with special reference to the type locality and bibliography. Nat. Hist. Res..

[19] Hwang M.S., Kim S., Ha D., Baek J.M., Kim H., Choi H. (2005). DNA sequences and identification of *Porphyra* cultivated by natural seeding on the southwest coast of Korea. Algae.

[20] Sun P., Mao Y., Li G., Cao M., Kong F., Wang L., Bi G. (2015). Comparative transcriptome profiling of *Pyropia yezoensis* (Ueda) M.S. Hwang & H.G. Choi in response to temperature stresses. BMC Genom..

[21] Noda H. (1993). Health benefits and nutritional properties of nori. J. Appl. Phycol..

[22] Park S.J., Ryu J.A., Kim I.H., Choi Y.H., Nam T.J. (2015). Activation of the mTOR signaling pathway in breast cancer MCF-7 cells by a peptide derived from *Porphyra yezoensis*. Oncol. Rep..

[23] Shin E.S., Hwang H.J., Kim I.H., NAM T.J. (2011). A glycoprotein from *Porphyra yezoensis* produces anti-inflammatory effects in liposaccharide-stimulated macrophages via the TLR4 signaling pathway. Int. J. Mol. Med..

[24] Ryu J.A., Park S.J., Kim I.H., Choi Y.H., Nam T.J. (2014). Protective effect porphyra-334 on UVA-induced photoaging in human skin fibroblasts. Int. J. Mol. Med..

[25] Tao C., Sugawara T., Maeda S., Wang X., Hirata T. (2008). Antioxidative activities of a mycosporine-like amino acid, porphyra-334. Fish. Sci..

[26] Choi J.W., Kim I.H., Kim Y.M., Lee M.K., Choi Y.H., Nam T.J. (2016). Protective effect of *Pyropia yezoensis* glycoprotein on chronic ethanol consumption-induced hepatotoxicity in rats. Mol. Med. Rep..

[27] Choi J.W., Kim Y.M., Park S.J., Kim I.H., Nam T.J. (2015). Protective effect of *Porphyra yezoensis* glycoprotein on d-galactosamine-induced cytotoxicity in Hepa 1c1c7 cells. Mol. Med. Rep..

[28] Hwang H.J., Kwon M.J., Kim I.H., Nam T.J. (2008). Chemoprotective effects protein from the red algae *Porphyra yezoensis* on acetaminophen-induced liver injury in rats. Phytother. Res..

[29] Lee M.K., Kim Y.M., Kim I.H., Choi Y.H., Nam T.J. (2017). *Pyropia yezoensis* peptide PYP1-5 protects against dexamethasone-induced muscle atrophy through the downregulation of atrogin1/MAFbx and MuRF1 in mouse C2C12 myotubes. Mol. Med. Rep..

[30] Sundanese N.R., Gupta M., Kim G., Rajamohan S.B., Isbatan A., Gupta M.P. (2009). Sirt3 blocks the cardiac hypertrophic response by augmenting Foxo3a-dependent antioxidant defense mechanisms in mice. J. Clin. Investig..

[31] Wang D., Miller S.C., Liu X.M., Anderson B., Wang X.S., Goldring S.R. (2007). Novel dexamethasone-HPMA copolymer conjugate and its potential application in treatment of rheumatoid arthritis. Arthritis Res. Ther..

[32] Barnes P.J. (2002). Scientific rationale for inhaled combination therapy with long-acting beta2-agonists and corticosteroids. Eur. Respir. J..

[33] Barnes P.J., Adcock I.M. (1998). Transcription factors and asthma. Eur. Respir. J..

[34] Dardevet D., Sornet C., Taillandier D., Savary J., Attaix D., Grizard J. (1995). Sensitivity and protein turnover response to glucocorticoids are different in skeletal muscle from adult and old rats. Lack of regulation of the ubiquitin-proteasome proteolytic pathway in aging. J. Clin. Investig..

[35] Mitch W.E., Goldberg A.L. (1996). Mechanisms of muscle wasting. The role of the ubiquitin-proteasome pathway. N. Engl. J. Med..

[36] Ma Z., Zhong Z., Zheng Z., Shi X.M., Zhang W. (2014). Inhibition of glycogen synthase kinase-3β attenuates glucocorticoid-induced suppression of myogenic differentiation in vitro. PLoS ONE.

[37] Bruscoli S., Donato V., Velardi E., Di Sante M., Migliorati G., Donato R., Riccardi C. (2010). Glucocorticoid-induced leucine zipper (GILZ) and long GILZ inhibit myogenic differentiation and mediate anti-myogenic effects of glucocorticoids. J. Biol. Chem..

[38] Moresi V., Williams A.H., Meadows E., Flynn J.M., Potthoff M.J., McAnally J., Shelton J.M., Backs J., Klein W.H., Richardson J.A. (2010). Myogenin and class II HDACs control neurogenic muscle atrophy by inducing E3 ubiquitin ligases. Cell.

[39] Stitt T.N., Drujan D., Clarke B.A., Panaro F., Timofeyva Y., Kline W.O., Gonzalez M., Yancopoulos G.D., Glass D.J. (2004). The IGF-I/PI3K/Akt pathway prevents expression of muscle atrophy-induced ubiquitin ligases by inhibiting FoxO transcription factors. Mol. Cell.

[40] te Pas M.F., de Jong P.R., Verburg F.J. (2000). Glucocorticoid inhibition of C2C12 proliferation rate and differentiation capacity in relation to mRNA levels of the MRF gene family. Mol. Biol. Rep..

[41] Milan G., Romanello V., Pescatore F., Armani A., Palk J.H., Frasson L., Seydel A., Zhao J., Abraham R., Goldberg A.L. (2015). Regulation of autophagy and the ubiquitin-proteasome system by the FoxO transcriptional network during muscle atrophy. Nat. Commun..

[42] Allen D.L., Unterman T.G. (2007). Regulation of myostatin expression and myoblast differentiation by FoxO and SMAD transcription factors. Am. J. Physiol. Cell. Physiol..

[43] Kamei Y., Miura S., Suzuki M., Kai Y., Mizukami J., Taniguchi T., Mochida K., Hata T., Matsuda J., Aburatani H. (2004). Skeletal muscle FOXO1 (FKHR) transgenic mice have less skeletal muscle mass, down-regulated type I (slow twitch/red muscle) fiber genes, and impaired glycemic control. J. Biol. Chem..

[44] Sandri M., Sandri C., Gilbert A., Skurk C., Calabria E., Picard A., Walsh K., Schiaffino S., Lecker S.H., Goldberg A.L. (2004). FoxO transcription factors induce the atrophy-related ubiquitin ligase atrogin-1 and cause skeletal muscle atrophy. Cell.

[45] Lee S.H., Barton E.R., Sweeney H.L., Farrar R.P. (2004). Viral expression of insulin-like growth factor-I enhances muscle hypertrophy in resistance-trained rats. J. Appl. Physiol..

[46] Giron M.D., Vilchez J.D., Shreeram S., Salto R., Manzano M., Cabrera E., Campos N., Edens N.K., Rueda R., Lopez-Pedrosa J.M. (2015). β-Hydroxy-β-methylbutyrate (HMB) normalizes dexamethasone-induced autophagy-lysosomal pathway in skeletal muscle. PLoS ONE.

[47] Teixeira V.D., Filippin L.I., Xavier R.M. (2012). Mechanisms of muscle wasting in sarcopenia. Rev. Bras. Reumatol..

[48] Glass D.J. (2005). Skeletal muscle hypertrophy and atrophy signaling pathways. Int. J. Biochem. Cell Biol..

[49] Hasselgren P.O., Wray C., Mammen J. (2002). Molecular regulation of muscle cachexia: It may be more than the proteasome. Biochem. Biophys. Res. Commun..

[50] Hershko A., Ciechanover A. (1998). The ubiquitin system. Annu. Rev. Biochem..

[51] Bechet D., Tassa A., Taillandier D., Cornbaret L., Attaix D. (2005). Lysosomal proteolysis in skeletal muscle. Int. J. Biochem. Cell Biol..

[52] Tassa A., Roux M.P., Attaix D., Bechet D.M. (2003). Class III phosphoinositide 3-kinase-Beclin1 complex mediates the amino acid-dependent regulation of autophagy in C2C12 myotubes. Biochem. J..

[53] Troncose R., Paredes F., Parra V., Gatica D., Vasquez-Trincado C., Quiroga C., Bravo-Sagua R., Lopez-Crisosto C., Rodriguez A.E., Oyarzun A.P. (2014). Dexamethasone-induced autophagy mediates muscle atrophy through mitochondrial clearance. Cell Cycle.

[54] Menconi M., Gonnella P., Petkova V., Lecker S., Hasselgren P.O. (2008). Dexamethasone and corticosterone induce similar, but not identical, muscle wasting responses in cultured L6 and C2C12 myotubes. J. Cell. Biochem..

[55] Gonnella P., Alamdari N., Tizio S., Aversa Z., Petkova V., Hasselgren P.O. (2011). C/EBPβ regulates dexamethasone-induced muscle cell atrophy and expression of atrogin-1 and MuRF1. J. Cell. Biochem..

